# The 30-Year Anniversary of *Pediatric Neurology Briefs*

**DOI:** 10.15844/pedneurbriefs-30-1-1

**Published:** 2016-01

**Authors:** John J. Millichap, J. Gordon Millichap

**Affiliations:** 1 Division of Neurology, Ann & Robert H. Lurie Children's Hospital of Chicago, Chicago, IL; 2 Departments of Pediatrics and Neurology, Northwestern University Feinberg School of Medicine, Chicago, IL

**Keywords:** Neurology, Pediatrics, Child Development, Nervous System Diseases, Brain Diseases

## Abstract

Over the past 30 years, *Pediatric Neurology Briefs* (PNB) has been published monthly as a continuing education service designed to expedite and facilitate review of current medical literature concerning pediatric neurology.

Over the past 30 years, *Pediatric Neurology Briefs* (PNB) has been published monthly as a continuing education service designed to expedite and facilitate review of current medical literature concerning pediatric neurology. The journal was founded in 1987 by J. Gordon Millichap in London, England, while the founding editor was on sabbatical as honorary consultant at Great Ormond Street Hospital. PNB has reviewed and referenced articles from over 200 journals since inception. So far, PNB consists of 29 volumes with greater than 3300 articles, and includes over 10,000 citations referencing the works of approximately 28,000 scholarly authors. Prior to the advent of the internet, the Editor periodically compiled and published the PNB articles in book form with index, according to subject heading and in chronological order [1 – 3].

The past year has been very successful. Recall that PNB was relaunched as an open access, peer-reviewed, journal with an expanded editorial board in January 2015. PNB had a new website and content management system capable of organizing peer-review and providing improved indexing, DOI assignment, and online full-text article view. Digitization is ongoing with over ten years of back issues including over 1100 full text open access articles available on the journal website so far. In 2016, we are proud to announce that PNB was selected for inclusion in PubMed Central® (PMC). PMC is a free archive of biomedical and life sciences journal literature at the U.S. National Institutes of Health’s National Library of Medicine (NIH/NLM). In addition, articles published in PNB are searchable on PubMed (http://www.ncbi.nlm.nih.gov/pubmed/).

Also in 2015, John J. Millichap became the journal Editor and continues to be supported by J. Gordon Millichap, as Founding Editor, the Editorial Advisory Board, and invited expert Contributing Editors. The Editors of PNB select source articles using criteria that include recent publication in a peer-reviewed journal and a topic of clinical value to practicing pediatric neurologists. PNB covers a comprehensive group of subject areas related to progress in pediatric neurology with notable additions in 2015 including Complementary/Integrative Therapies [4]. Contributing Editors provide detailed summaries of published articles, followed by commentaries based on their experience and corroborated by appropriate supplementary citations. The two most highly accessed articles published in 2015 covered topics related to epilepsy including the mechanisms of the ketogenic diet [5] and the history of the specific EEG term, “hypsarhythmia” [6].

The mission of PNB remains the same: “Pediatric Neurology Briefs is a continuing education service designed to expedite and facilitate the review of current scientific research and advances in child neurology and related subjects.” The Editors of PNB endeavor to deliver a ‘clinical pearl’ with each commentary that will be of interest and use in clinical practice. New in 2016, topic experts interested in contributing to PNB as authors or reviewers are invited to contact the Editor at j-millichap@northwestern.edu.
